# A Hospital for Poor Mothers

**Published:** 1912-07-20

**Authors:** 


### A HOSPITAL FOR POOR MOTHERS.

If it be true that troubles never come singly, it frequently happens that boons also arrive in quick succession. Two notable efforts to ameliorate the conditions of maternity among the very poor have recently illustrated this contention. To begin with, her Royal Highness the Princess Louise (Duchess of Argyll) laid the foundation-stone on July 4 of a Mothers' Hospital in the Lower Clapton Road, organised mainly by Mrs. Bramwell Booth. The hospital represents an extension of and improvement upon the efforts originally started by the Salvation Army for unmarried mothers. In the new hospital married as well as unmarried women will be received, and it is hoped eventually to provide accommodation for one hundred at a time. So far the funds in hand permit of premises for only forty-eight, but the site obtained will allow of further extensions as they may be provided for. At present four bungalow wards are being erected, each capable of accommodating twelve patients. One will contain exclusively married mothers, one unmarried mothers, and one Jewish mothers; the fourth is reserved for special cases. The land secured consists of about three acres, with a façade of six semi-detached houses. On so extensive (for London) a site it should be possible to construct a splendidly open and airy series of pavilions.

The other announcement of help for poor mothers is contained in the will of the late Mr. H. S. Trower, published last week, whereby he devises £10,000 to four trustees to form a Women's Aid Fund. The objects of the testator are to assist pregnant married or unmarried women with medical care and nursing before, during, and after confinement. He was especially desirous that expecting mothers should have skilled care in the critical weeks before delivery and afterwards until sufficiently recovered to resume their avocations. As regards such care during the last few weeks of the gestation period, there are difficulties to face. It is not only unnecessary and undesirable to afford poor mothers institutional care at that juncture, but it is actually almost impossible. Every physician knows the difficulty of accurately forecasting the date of delivery, even with a carefully-kept history to guide him; and among the very poor the untrustworthiness of menstrual history is a commonplace with officers of lying-in hospitals. So that any scheme to give mothers-elect a fortnight's or a month's rest in a hospital is bound to meet many disappointments. Nor is such excess of care really advisable, for it would of necessity deprive the patients of a good deal of exercise; the household duties which poor women have to carry on right up to confinement are often a blessing in disguise by endowing them with vigour and vitality for their ordeal such as many in better circumstances are found to lack.

These two philanthropic enterprises will be seen to have much in common. They may be taken to denote an awakening of the national conscience in respect of some of the problems of maternity and mothering. In both cases the after-care of recently delivered mothers, and the no less important preliminary care of those who are expectant, are prominent features of the enterprises. In both cases the benefits of the charity are to be extended without question of the civil status of the women. Many of our existing maternity charities will accept unmarried mothers only when they have had no previous experience of the ordeal of maternity. This condition may work occasional hardship, but it is easy to see that it is in many respects right and necessary. A lying-in hospital can admit only such patients to its wards as there are room and funds for. Thus, when every unmarried mother admitted keeps out a married one it is only right that those who fall more than once should give place to those who have not fallen at all. In neither of the two schemes above mentioned is this point raised, but we have no doubt it will receive the very careful consideration of the committees in charge of the arrangements. The two schemes should prove of immense value to a class than whom none deserve better the assistance of such charities as the layman who has read Mr. George Moore's "Esther Waters" will easily realise. The risk of overlapping of similar agencies should be in both cases easily avoided: in the one by the experience and organisation of the Salvation Army officers; in the other by the presence on the administering committee of Mr. Frederick Morris, secretary of the Marylebone branch of the Charity Organisation Society.

